# Correction: The fall—And rise—In hospital-based care for people with HIV in South Africa: 2004–2017

**DOI:** 10.1371/journal.pgph.0005169

**Published:** 2025-09-08

**Authors:** Evelyn Lauren, Khumbo Shumba, Matthew P. Fox, William MacLeod, Wendy Stevens, Koleka Mlisana, Jacob Bor, Dorina Onoya

The affiliation for the fifth author is incorrect. The correct affiliation is not indicated. Wendy Stevens affiliated with Wits Diagnostics Innovation Hub, Health Sciences Research Office, Faculty of Health Sciences, University of the Witwatersrand, Johannesburg, South Africa.
