# Supplementary material for: The fall—And rise—In hospital-based care for people with HIV in South Africa: 2004–2017
Source: PLOS Glob Public Health. 2024 Sep 5;4(9):e0002127. doi: 10.1371/journal.pgph.0002127 (PMC11376578; doi:10.1371/journal.pgph.0002127)
Supplement: S1 Checklist — (DOCX) [file pgph.0002127.s001.docx]

STROBE Statement—checklist of items that should be included in reports of observational studies

|  | Item No. | Recommendation | Page  No. | Relevant text from manuscript |
| --- | --- | --- | --- | --- |
| **Title and abstract** | 1 | (*a*) Indicate the study’s design with a commonly used term in the title or the abstract | 2,3 | Study design is indicated in methods/results section in the abstract.  “We assessed trends in hospital-based HIV care using a novel database: the National Health Laboratory Service (NHLS) National HIV Cohort.” (Page 2 Line 4) |
|  |  | (*b*) Provide in the abstract an informative and balanced summary of what was done and what was found | 2 | Summary was indicated in the methods/results section in the abstract.  Summary of what was done can be found in the methods section in the abstract (Page 2 Line 4-12)  Summary of what was found can be found in the results section in the abstract  (Page 2 Line 13-19) |
| Introduction | | | |  |
| Background/rationale | 2 | Explain the scientific background and rationale for the investigation being reported | 3 | Background and rationale are stated in the introduction section of the paper (Page 3 Line 2-25) |
| Objectives | 3 | State specific objectives, including any prespecified hypotheses | 3 | A statement at the end of the introduction specifies the specific goals and objectives.  (Page 3 Line 19-25) |
| Methods | | | |  |
| Study design | 4 | Present key elements of study design early in the paper | 3-4 | Key elements of study design are all described in the methods section.  “longitudinal patient-level analysis of all lab-monitored patients in the public sector HIV program” (Page 4 Line 17-18) |
| Setting | 5 | Describe the setting, locations, and relevant dates, including periods of recruitment, exposure, follow-up, and data collection | 4 | These are located in the “Data sources” and “Study population” on page 4 line 1-29. |
| Participants | 6 | (*a*) *Cohort study*—Give the eligibility criteria, and the sources and methods of selection of participants. Describe methods of follow-up  *Case-control study*—Give the eligibility criteria, and the sources and methods of case ascertainment and control selection. Give the rationale for the choice of cases and controls  *Cross-sectional study*—Give the eligibility criteria, and the sources and methods of selection of participants | 4 | These are located in the “Data sources” and “Study population” on page 4 line 1-29. |
|  |  | (*b*) *Cohort study*—For matched studies, give matching criteria and number of exposed and unexposed  *Case-control study*—For matched studies, give matching criteria and the number of controls per case | N/A | N/A |
| Variables | 7 | Clearly define all outcomes, exposures, predictors, potential confounders, and effect modifiers. Give diagnostic criteria, if applicable | 4-7 | These are located under the “Measures” and “Analyses” subsection (Page 4 line 30 – Page 7 line 5). |
| Data sources/ measurement | 8* | For each variable of interest, give sources of data and details of methods of assessment (measurement). Describe comparability of assessment methods if there is more than one group | 4-7 | Data collection and measurement was the same for all variables, as described in the methods section (page 4 line 1 – page 7 line 5). |
| Bias | 9 | Describe any efforts to address potential sources of bias | 4-7 | We excluded some data that might lead to bias.  “To maintain consistency, KwaZulu-Natal was excluded from all national-level analyses, because the province joined NHLS in 2010.” (Page 4 line 27-29).  “Lab tests at other facilities such as prisons, military bases, and psychiatric facilities were excluded.” (page 4 line 24-25) |
| Study size | 10 | Explain how the study size was arrived at | 4 | The study population subsection describes the inclusion/exclusion criteria to arrive at the study population (page 4 line 22-29). |

Continued on next page

| Quantitative variables | 11 | Explain how quantitative variables were handled in the analyses. If applicable, describe which groupings were chosen and why | 4-5 | Definitions of quantitative variables used in the analysis was located in the “Measures” subsection. (Page 4 line 30 – page 5 line 21) |
| --- | --- | --- | --- | --- |
| Statistical methods | 12 | (*a*) Describe all statistical methods, including those used to control for confounding | 5-7 | Definitions of statistical methods used and controls for confounding were described in the “Analyses” subsection (Page 5 line 23 – Page 7 line 5) |
|  |  | (*b*) Describe any methods used to examine subgroups and interactions | 5-7 | Description of methods used to examine subgroups were located in the “Analyses” subsection (Page 5 line 23 – Page 7 line 5).  Interactions not applicable. |
|  |  | (*c*) Explain how missing data were addressed | NA | There was an insignificant amount of missing data in our analysis and hence it is not addressed. |
|  |  | (*d*) *Cohort study*—If applicable, explain how loss to follow-up was addressed  *Case-control study*—If applicable, explain how matching of cases and controls was addressed  *Cross-sectional study*—If applicable, describe analytical methods taking account of sampling strategy | NA | Not applicable for this analysis |
|  |  | (*e*) Describe any sensitivity analyses | NA | Not applicable |
| Results | | | | |
| Participants | 13* | (a) Report numbers of individuals at each stage of study—eg numbers potentially eligible, examined for eligibility, confirmed eligible, included in the study, completing follow-up, and analysed | 7 | Number of individuals are located in the first paragraph of the results section, page 7 line 13-18. There were only one stage (all eligible patients were analyzed) |
|  |  | (b) Give reasons for non-participation at each stage | NA | All eligible patients were analyzed, therefore reason for non-participation is not applicable. |
|  |  | (c) Consider use of a flow diagram | NA | Use of flow diagram was not deemed appropriate |
| Descriptive data | 14* | (a) Give characteristics of study participants (eg demographic, clinical, social) and information on exposures and potential confounders | 7-9 | Characteristics of study participants and information on exposures and confounders were included when analyzing the models |
|  |  | (b) Indicate number of participants with missing data for each variable of interest | NA | Not applicable. |
|  |  | (c) *Cohort study*—Summarise follow-up time (eg, average and total amount) | 7-9 | Information on follow-up were included throughout the results section. |
| Outcome data | 15* | *Cohort study*—Report numbers of outcome events or summary measures over time | *7-9* | Outcome and summary measures were included in the results section (page 7 line 12 – page 9 line 30) |
|  |  | *Case-control study—*Report numbers in each exposure category, or summary measures of exposure |  |  |
|  |  | *Cross-sectional study—*Report numbers of outcome events or summary measures |  |  |
| Main results | 16 | (*a*) Give unadjusted estimates and, if applicable, confounder-adjusted estimates and their precision (eg, 95% confidence interval). Make clear which confounders were adjusted for and why they were included | *7-9* | These were included in the results section (page 7 line 12 – page 9 line 30) |
|  |  | (*b*) Report category boundaries when continuous variables were categorized | *7-9* | These were included in the results section (page 7 line 12 – page 9 line 30) |
|  |  | (*c*) If relevant, consider translating estimates of relative risk into absolute risk for a meaningful time period | *7-9* | These were included in the results section (page 7 line 12 – page 9 line 30) |

Continued on next page

| Other analyses | 17 | Report other analyses done—eg analyses of subgroups and interactions, and sensitivity analyses | *7-9* | These were included in the results section (page 7 line 12 – page 9 line 30) |
| --- | --- | --- | --- | --- |
| Discussion | | | | |
| Key results | 18 | Summarise key results with reference to study objectives | 9-10 | These were located in the first paragraph of the discussion section (page 9 line 33 – page 10 line 2) |
| Limitations | 19 | Discuss limitations of the study, taking into account sources of potential bias or imprecision. Discuss both direction and magnitude of any potential bias | 11 | These were located in the last paragraph of the discussion section (page 11 line10 -21). |
| Interpretation | 20 | Give a cautious overall interpretation of results considering objectives, limitations, multiplicity of analyses, results from similar studies, and other relevant evidence | 10-11 | These were located throughout the discussion section (page 10 line 3 – page 11 line 8) |
| Generalisability | 21 | Discuss the generalisability (external validity) of the study results | 10-11 | These were located throughout the discussion section (page 10 line 3 – page 11 line 8) |
| Other information | |  | | |
| Funding | 22 | Give the source of funding and the role of the funders for the present study and, if applicable, for the original study on which the present article is based | 2 | This is located under the funding section in page 2 lines 24-26. |

*Give information separately for cases and controls in case-control studies and, if applicable, for exposed and unexposed groups in cohort and cross-sectional studies.

**Note:** An Explanation and Elaboration article discusses each checklist item and gives methodological background and published examples of transparent reporting. The STROBE checklist is best used in conjunction with this article (freely available on the Web sites of PLoS Medicine at http://www.plosmedicine.org/, Annals of Internal Medicine at http://www.annals.org/, and Epidemiology at http://www.epidem.com/). Information on the STROBE Initiative is available at www.strobe-statement.org.
