# Supplementary material for: The fall—And rise—In hospital-based care for people with HIV in South Africa: 2004–2017
Source: PLOS Glob Public Health. 2024 Sep 5;4(9):e0002127. doi: 10.1371/journal.pgph.0002127 (PMC11376578; doi:10.1371/journal.pgph.0002127)
Supplement: S1 Table — (DOCX) [file pgph.0002127.s002.docx]

**S1 Table. Risk ratio of hospitalization in 2 years after presentation by year of entry to care**

|  | Unadjusted model | Adjusted for age, sex, province | Adjusted for age, sex, province, facility type | Adjusted for age, sex, province, CD4 count at entry | Adjusted for age, sex, province, facility type, CD4 count at entry |
| --- | --- | --- | --- | --- | --- |
| Year of entry to care | Risk ratio (95% CI) | Risk ratio (95% CI) | Risk ratio (95% CI) | Risk ratio (95% CI) | Risk ratio (95% CI) |
| 2004 | ref. | ref. | ref. | ref. | ref. |
| 2005 | 0.96 (0.95, 0.98) | 0.96 (0.94, 0.98) | 1.01 (1.00, 1.03) | 0.99 (0.98, 1.01) | 1.04 (1.02, 1.06) |
| 2006 | 0.86 (0.84, 0.87) | 0.85 (0.84, 0.87) | 0.97 (0.95, 0.98) | 0.89 (0.88, 0.91) | 1.00 (0.98, 1.01) |
| 2007 | 0.80 (0.78, 0.81) | 0.80 (0.78, 0.81) | 0.94 (0.92, 0.95) | 0.85 (0.83, 0.86) | 0.97 (0.96, 0.99) |
| 2008 | 0.78 (0.76, 0.79) | 0.77 (0.76, 0.79) | 0.94 (0.93, 0.96) | 0.82 (0.81, 0.84) | 0.98 (0.96, 0.99) |
| 2009 | 0.70 (0.69, 0.71) | 0.70 (0.68, 0.71) | 0.87 (0.85, 0.88) | 0.76 (0.74, 0.77) | 0.91 (0.90, 0.93) |
| 2010 | 0.62 (0.61, 0.63) | 0.61 (0.60, 0.62) | 0.78 (0.77, 0.80) | 0.67 (0.66, 0.69) | 0.83 (0.82, 0.85) |
| 2011 | 0.55 (0.54, 0.56) | 0.54 (0.53, 0.55) | 0.70 (0.69, 0.71) | 0.61 (0.60, 0.62) | 0.76 (0.75, 0.77) |
| 2012 | 0.53 (0.52, 0.54) | 0.53 (0.52, 0.54) | 0.69 (0.68, 0.71) | 0.60 (0.59, 0.61) | 0.76 (0.75, 0.77) |
| 2013 | 0.54 (0.53, 0.55) | 0.54 (0.53, 0.55) | 0.71 (0.70, 0.73) | 0.63 (0.61, 0.64) | 0.79 (0.78, 0.81) |
| 2014 | 0.57 (0.56, 0.58) | 0.57 (0.56, 0.58) | 0.75 (0.74, 0.77) | 0.66 (0.64, 0.67) | 0.84 (0.82, 0.85) |
| 2015 | 0.63 (0.62, 0.64) | 0.63 (0.62, 0.64) | 0.84 (0.83, 0.86) | 0.73 (0.72, 0.74) | 0.94 (0.92, 0.96) |
