# Supplementary material for: The fall—And rise—In hospital-based care for people with HIV in South Africa: 2004–2017
Source: PLOS Glob Public Health. 2024 Sep 5;4(9):e0002127. doi: 10.1371/journal.pgph.0002127 (PMC11376578; doi:10.1371/journal.pgph.0002127)
Supplement: S2 Table — (DOCX) [file pgph.0002127.s003.docx]

**S2 Table. Risk ratio of hospitalization in 2 years after viral suppression by year of entry to care**

|  | Unadjusted model | Adjusted for age, sex, province | Adjusted for age, sex, province, facility type | Adjusted for age, sex, province, CD4 count at entry | Adjusted for age, sex, province, facility type, CD4 count at entry |
| --- | --- | --- | --- | --- | --- |
| Year of entry to care | Risk ratio (95% CI) | Risk ratio (95% CI) | Risk ratio (95% CI) | Risk ratio (95% CI) | Risk ratio (95% CI) |
| 2004 | ref. | ref. | ref. | ref. | ref. |
| 2005 | 0.97 (0.95, 0.99) | 0.95 (0.92, 0.97) | 0.98 (0.96, 1.00) | 0.96 (0.94, 0.98) | 1.00 (0.97, 1.02) |
| 2006 | 0.79 (0.77, 0.81) | 0.76 (0.74, 0.78) | 0.83 (0.81, 0.85) | 0.77 (0.76, 0.79) | 0.85 (0.83, 0.87) |
| 2007 | 0.72 (0.70, 0.73) | 0.69 (0.67, 0.71) | 0.78 (0.76, 0.80) | 0.71 (0.70, 0.73) | 0.80 (0.78, 0.82) |
| 2008 | 0.67 (0.65, 0.68) | 0.64 (0.62, 0.65) | 0.74 (0.72, 0.75) | 0.65 (0.64, 0.67) | 0.76 (0.74, 0.77) |
| 2009 | 0.53 (0.52, 0.55) | 0.51 (0.50, 0.52) | 0.60 (0.58, 0.61) | 0.53 (0.52, 0.55) | 0.63 (0.61, 0.64) |
| 2010 | 0.42 (0.41, 0.43) | 0.41 (0.40, 0.42) | 0.50 (0.49, 0.51) | 0.43 (0.42, 0.44) | 0.53 (0.52, 0.54) |
| 2011 | 0.34 (0.34, 0.35) | 0.33 (0.33, 0.34) | 0.43 (0.42, 0.44) | 0.36 (0.35, 0.37) | 0.47 (0.45, 0.48) |
| 2012 | 0.30 (0.29, 0.31) | 0.29 (0.28, 0.29) | 0.38 (0.37, 0.38) | 0.32 (0.31, 0.33) | 0.42 (0.41, 0.43) |
| 2013 | 0.29 (0.29, 0.30) | 0.28 (0.28, 0.29) | 0.38 (0.37, 0.39) | 0.32 (0.32, 0.33) | 0.43 (0.42, 0.44) |
| 2014 | 0.31 (0.30, 0.31) | 0.29 (0.29, 0.30) | 0.39 (0.38, 0.40) | 0.34 (0.33, 0.35) | 0.45 (0.44, 0.46) |
| 2015 | 0.36 (0.35, 0.37) | 0.34 (0.34, 0.35) | 0.45 (0.44, 0.47) | 0.41 (0.40, 0.42) | 0.54 (0.52, 0.55) |
