# Supplementary material for: The fall—And rise—In hospital-based care for people with HIV in South Africa: 2004–2017
Source: PLOS Glob Public Health. 2024 Sep 5;4(9):e0002127. doi: 10.1371/journal.pgph.0002127 (PMC11376578; doi:10.1371/journal.pgph.0002127)
Supplement: S3 Table — (DOCX) [file pgph.0002127.s004.docx]

**S3 Table. Potential factors driving hospitalization rates**

| Year of entry to care | # tests done | # patients with tests | # CD4 tests | # VL tests | # ALT tests | # CRT tests | # HG tests |
| --- | --- | --- | --- | --- | --- | --- | --- |
| 2004 | 651,439 | 133,690 | 264,425 | 58,703 | 109,049 | 129 | 158,456 |
| 2005 | 1,651,245 | 333,659 | 649,654 | 171,556 | 264,467 | 227 | 371,986 |
| 2006 | 2,347,162 | 459,924 | 900,931 | 268,155 | 379,266 | 259 | 498,874 |
| 2007 | 2,742,532 | 529,471 | 1,026,599 | 326,163 | 461,959 | 363 | 593,269 |
| 2008 | 3,366,671 | 646,625 | 1,248,059 | 394,888 | 580,894 | 8,011 | 740,100 |
| 2009 | 3,310,373 | 650,451 | 1,245,236 | 316,662 | 558,871 | 101,394 | 732,731 |
| 2010 | 4,054,576 | 765,016 | 1,463,247 | 346,221 | 659,119 | 427,383 | 848,065 |
| 2011 | 4,080,924 | 749,670 | 1,368,610 | 372,013 | 644,413 | 716,081 | 811,460 |
| 2012 | 3,678,218 | 661,365 | 1,148,957 | 385,988 | 545,355 | 777,713 | 703,585 |
| 2013 | 3,292,653 | 553,839 | 960,781 | 386,691 | 403,667 | 856,343 | 572,892 |
| 2014 | 3,294,514 | 525,245 | 874,555 | 449,118 | 339,388 | 983,589 | 529,110 |
| 2015 | 3,545,886 | 527,246 | 855,091 | 556,828 | 313,085 | 1,161,253 | 528,879 |

| Year of entry to care | # hospital-lizations | # hosp GP | # hosp WC | # hosp all other prov | # patients with any hosp | # patients with 1 hosp | # patients with >1 |
| --- | --- | --- | --- | --- | --- | --- | --- |
| 2004 | 22,596 | 7,217 | 4,088 | 11,335 | 15,901 | 11,640 | 10,956 |
| 2005 | 52,643 | 16,391 | 5,932 | 30,453 | 37,665 | 27,911 | 24,732 |
| 2006 | 61,681 | 17,251 | 5,779 | 38,844 | 46,606 | 35,976 | 25,705 |
| 2007 | 66,616 | 17,772 | 6,117 | 42,915 | 49,878 | 38,478 | 28,138 |
| 2008 | 79,354 | 20,000 | 6,924 | 52,668 | 59,418 | 45,510 | 33,844 |
| 2009 | 69,733 | 17,932 | 7,154 | 44,855 | 54,175 | 42,970 | 26,763 |
| 2010 | 71,742 | 19,848 | 8,216 | 43,871 | 56,523 | 45,842 | 25,900 |
| 2011 | 61,812 | 18,351 | 7,238 | 36,415 | 48,653 | 39,600 | 22,212 |
| 2012 | 52,709 | 16,465 | 5,792 | 30,611 | 40,904 | 32,930 | 19,779 |
| 2013 | 48,585 | 16,118 | 5,004 | 27,620 | 36,983 | 29,419 | 19,166 |
| 2014 | 49,527 | 17,169 | 5,010 | 27,518 | 37,141 | 29,221 | 20,306 |
| 2015 | 56,114 | 19,930 | 4,934 | 31,408 | 41,231 | 31,801 | 24,313 |

| Year of entry to care | # hospitals | # clinics |
| --- | --- | --- |
| 2004 | 291 | 1,944 |
| 2005 | 308 | 2,692 |
| 2006 | 312 | 3,003 |
| 2007 | 314 | 3,091 |
| 2008 | 322 | 3,157 |
| 2009 | 321 | 3,274 |
| 2010 | 327 | 3,788 |
| 2011 | 334 | 4,128 |
| 2012 | 334 | 4,056 |
| 2013 | 351 | 3,915 |
| 2014 | 366 | 3,584 |
| 2015 | 369 | 3,537 |
