# Supplementary figures and images for: The fall—And rise—In hospital-based care for people with HIV in South Africa: 2004–2017
Source: PLOS Glob Public Health. 2024 Sep 5;4(9):e0002127. doi: 10.1371/journal.pgph.0002127 (PMC11376578; doi:10.1371/journal.pgph.0002127)

**S1 Fig. Number of patients presenting to HIV care by facility and province**


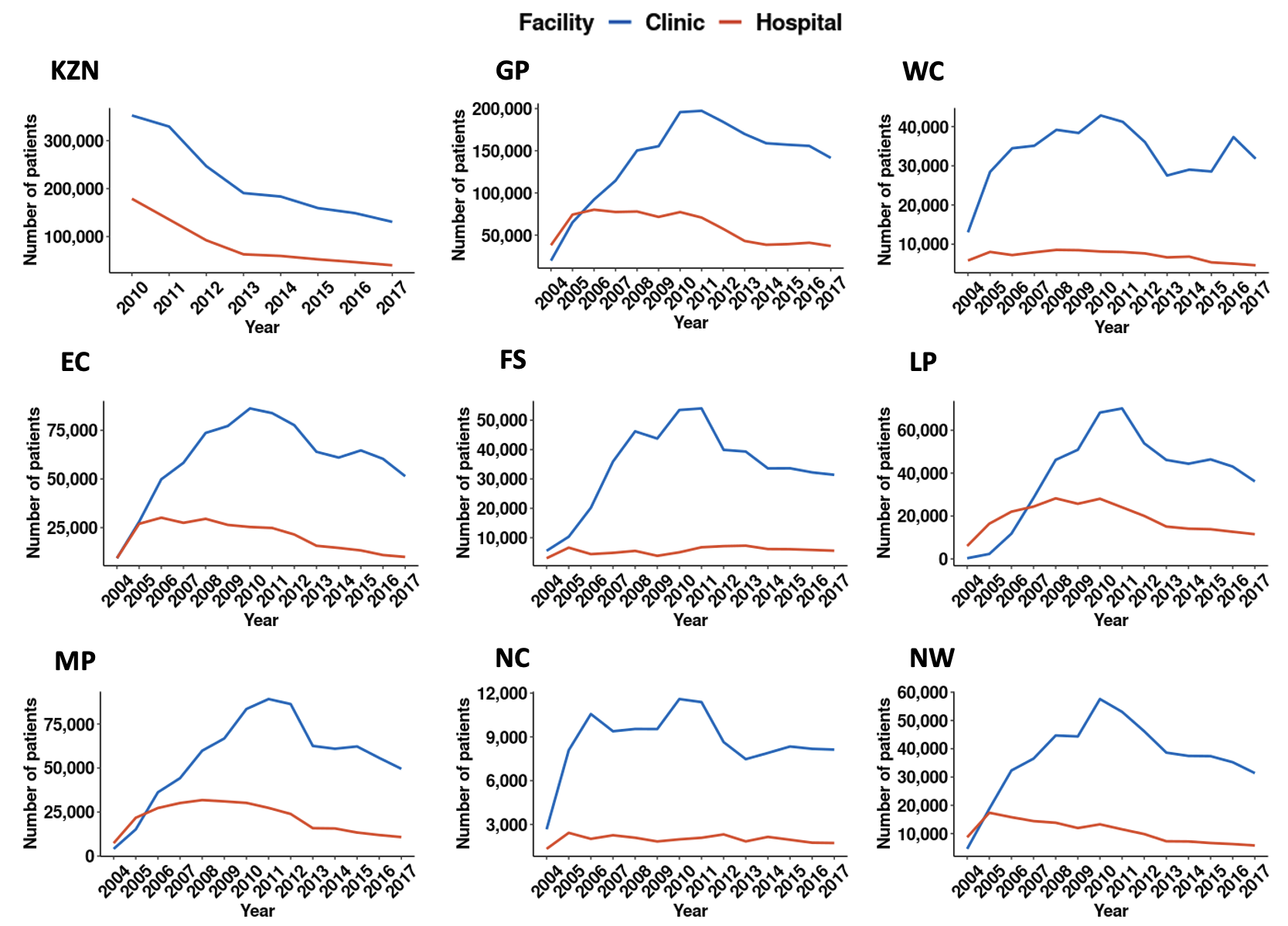

Supplement: S1 Fig — (DOCX) [file pgph.0002127.s005.docx]

**S2 Fig. Number of patients receiving HIV care by facility and province**


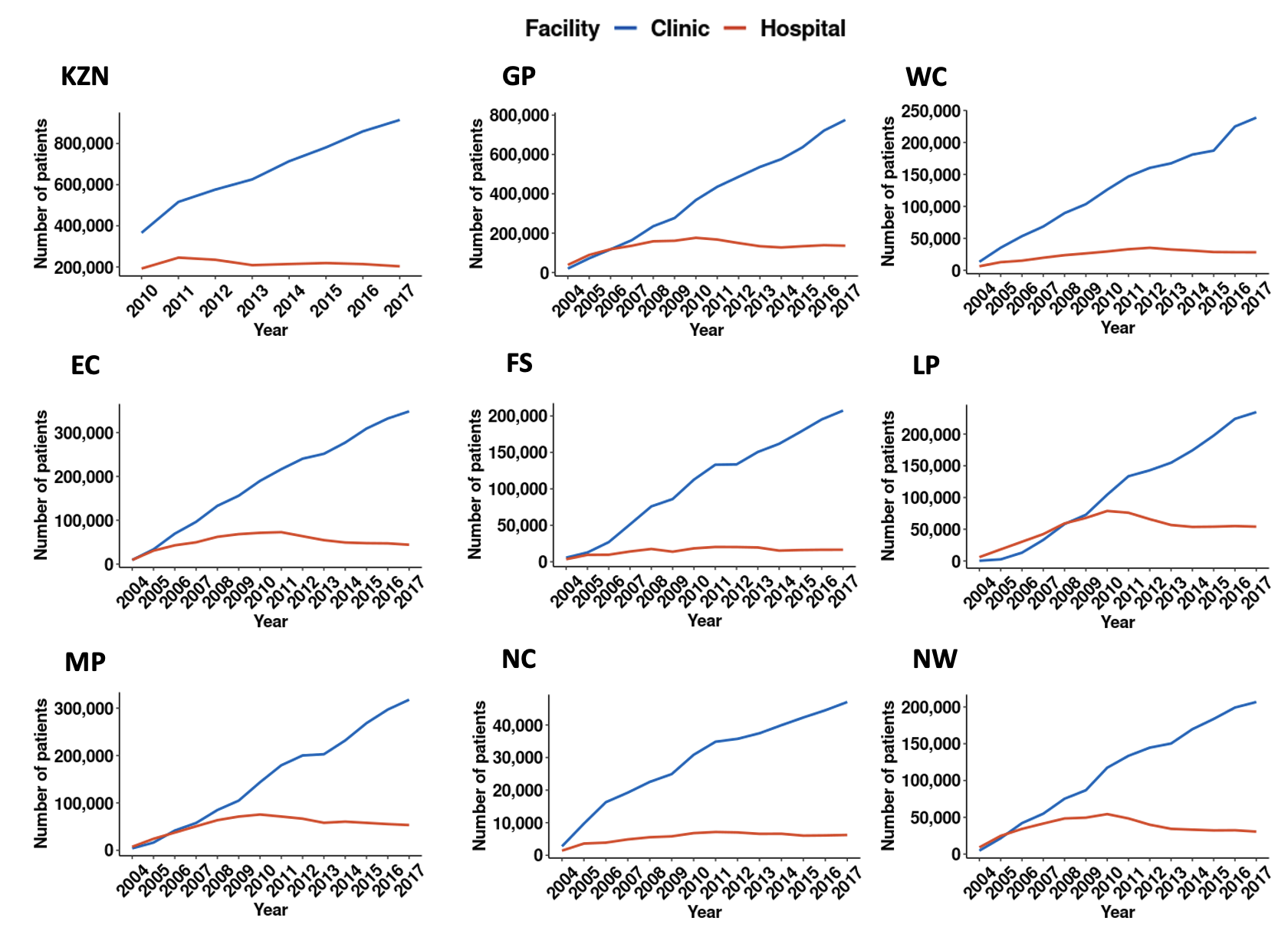

Supplement: S2 Fig — (DOCX) [file pgph.0002127.s006.docx]

**S5 Fig. Percentage of patients hospitalized within 2 years after presentation by age group (18-39, 40-64, 65+)**

**
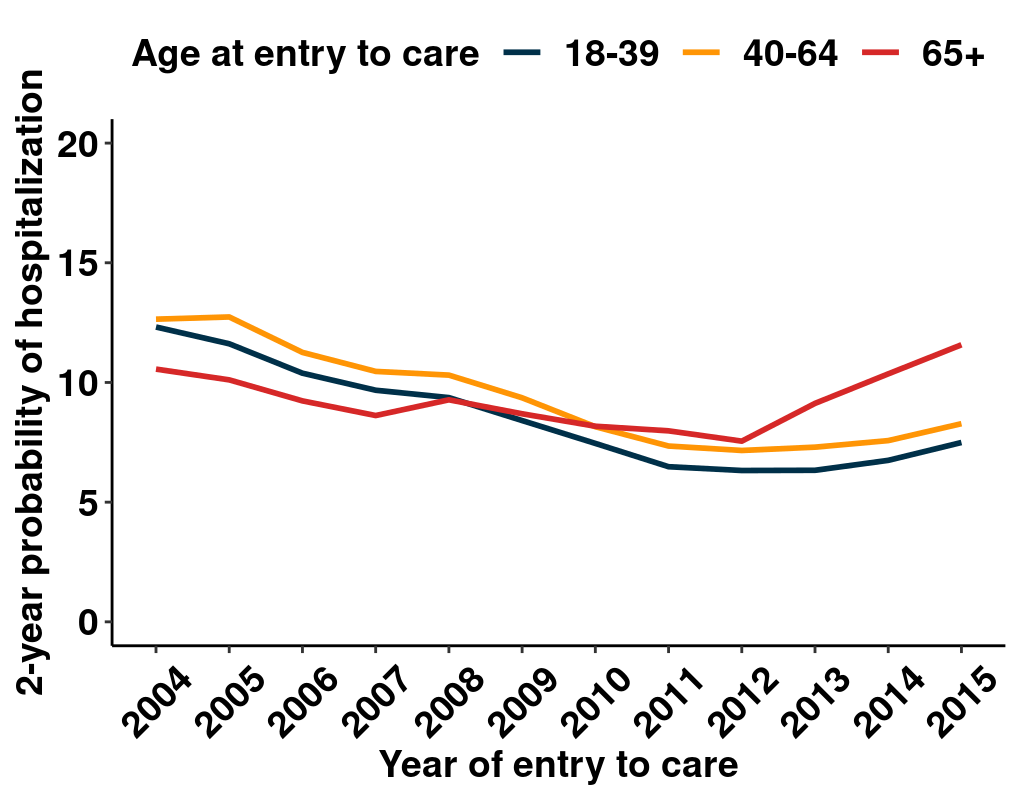
**

Supplement: S5 Fig — (DOCX) [file pgph.0002127.s009.docx]
