# Supplementary material for: The fall—And rise—In hospital-based care for people with HIV in South Africa: 2004–2017
Source: PLOS Glob Public Health. 2024 Sep 5;4(9):e0002127. doi: 10.1371/journal.pgph.0002127 (PMC11376578; doi:10.1371/journal.pgph.0002127)
Supplement: S3 Fig — (DOCX) [file pgph.0002127.s007.docx]

**S3 Fig. Trends in lab test results at first hospitalization**


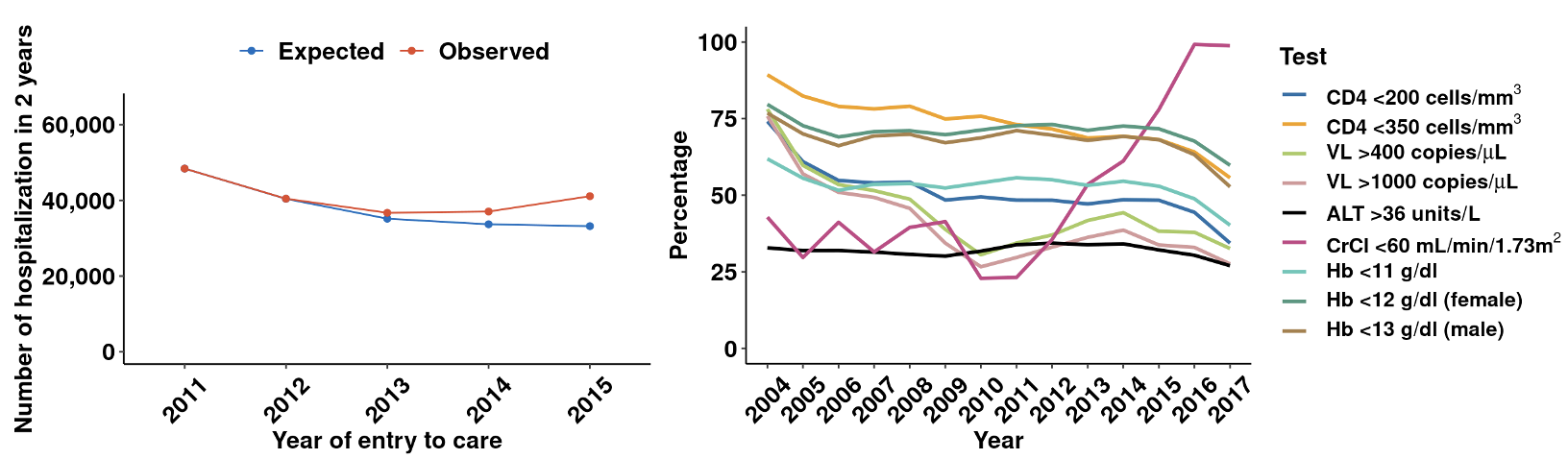


**Note:** Figure shows the percentage of patients with a laboratory test result indicating poor health at the time of hospitalization. Laboratory test definitions and clinical thresholds are provided in Table 2. Hb <12 g/dl indicates moderate-to-severe anemia in non-pregnant women, and Hb <13 g/dl indicates moderate-to-severe anemia in men.
