# Supplementary material for: The fall—And rise—In hospital-based care for people with HIV in South Africa: 2004–2017
Source: PLOS Glob Public Health. 2024 Sep 5;4(9):e0002127. doi: 10.1371/journal.pgph.0002127 (PMC11376578; doi:10.1371/journal.pgph.0002127)

**S4 Fig. Percentage of patients hospitalized within 2 years after presentation and viral suppression by facility at entry to care**


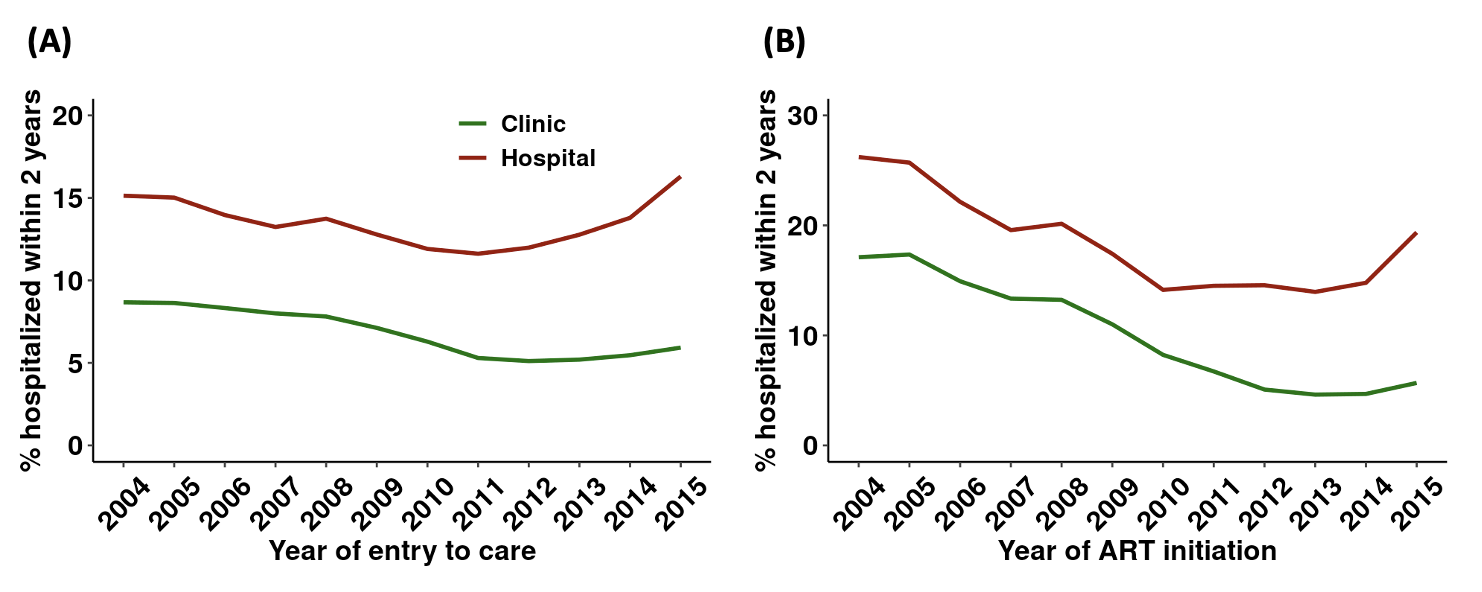

Supplement: S4 Fig — (DOCX) [file pgph.0002127.s008.docx]
