# Supplementary material for: The fall—And rise—In hospital-based care for people with HIV in South Africa: 2004–2017
Source: PLOS Glob Public Health. 2024 Sep 5;4(9):e0002127. doi: 10.1371/journal.pgph.0002127 (PMC11376578; doi:10.1371/journal.pgph.0002127)
Supplement: Attachment — Submitted filename: Response to reviewers.docx [file pgph.0002127.s010.docx]

Response to reviewers

Dear PLOS Global Public Health Editor,

There were no editor and reviewer comments included in the decision letter of the manuscript. Hence, as requested by the journal, we had addressed the journal requirements and are resubmitting our paper back to the office for further checking.

Thank you,

Evelyn Lauren
