# Supplementary material for: The fall—And rise—In hospital-based care for people with HIV in South Africa: 2004–2017
Source: PLOS Glob Public Health. 2024 Sep 5;4(9):e0002127. doi: 10.1371/journal.pgph.0002127 (PMC11376578; doi:10.1371/journal.pgph.0002127)
Supplement: Attachment — Submitted filename: response_to_reviewers_v3.docx [file pgph.0002127.s012.docx]

August 6, 2024

Attn: Marianne Clemence, DPhil

Staff Editor, PLOS Global Public Health

**Re: The fall -- and rise -- in hospital-based care for people with HIV in South Africa: 2004-2017**

To Dr. Clemence:

Thank you for the opportunity to revise and resubmit our manuscript, “The fall -- and rise -- in hospital-based care for people with HIV in South Africa: 2004-2017,” for consideration at *PLOS Global Public Health*.

We have attached detailed responses to the reviewers’ comments below. We believe the feedback has strengthened the manuscript and welcome further editorial suggestions.

Thank you again for your consideration of our manuscript.

Sincerely,

Jacob Bor ([jbor@bu.edu](mailto:jbor@bu.edu))

**Review Comments to the Author**

***Reviewer #1:*** *Dear Authors, Thank you for your robust responses to my comments. I am satisfied that all my comments have been answered. I have no further comments.*

**Response:** Thank you for taking the time to review the manuscript. We highly appreciate all the comments and feedback.

***Reviewer #2:*** *Thank you for the opportunity to review the revised manuscript. The authors did a good job addressing the comments I had. A few minor things to consider*

*i) NICD in line 136 in the revised manuscript should be National Institute for Communicable Diseases and not clinical diseases*

**Response:** Thank you for the comment. We now write “National Institute for Communicable Diseases” on line 129 of the manuscript.

*ii) in the comparison 18- 39 vs 65+, why was 40- 64 left out of the discussion and supplementary chart?*

**Response:** Thank you for pointing this out. We focused on the age groups 18-39 and 65+ in the discussion to highlight the differences in temporal trends in hospitalization between the younger and older populations. Hospitalizations among younger individuals are more likely to be HIV-related, while hospitalizations among older adults are often due to reasons unrelated to HIV, such as age-related diseases.

In the discussion, we suggested that the observed decline in hospitalizations over time in younger adults but not among older adults may indicate a decline in HIV-related hospitalizations and a rise in hospitalizations due to other reasons (e.g., age-related factors).

However, we agree with the reviewer’s observation that there was no reason to exclude the 40-64 age group from the supplementary chart. We have now added a new line for 40-64 year olds to the supplementary figure S5.
