# Supplementary material for: The fall—And rise—In hospital-based care for people with HIV in South Africa: 2004–2017
Source: PLOS Glob Public Health. 2024 Sep 5;4(9):e0002127. doi: 10.1371/journal.pgph.0002127 (PMC11376578; doi:10.1371/journal.pgph.0002127)
Supplement: Attachment — Submitted filename: reviewer comments v2 JB.docx [file pgph.0002127.s011.docx]

June 1, 2024

Attn: Syed Shahid Abbas, MBBS, MPH, Ph.D.

Academic Editor, PLOS Global Public Health

**Re: The fall -- and rise -- in hospital-based care for people with HIV in South Africa: 2004-2017**

To Dr. Abbas:

Thank you for the opportunity to revise and resubmit our manuscript, “The fall -- and rise -- in hospital-based care for people with HIV in South Africa: 2004-2017,” for consideration at *PLOS Global Public Health*.

We have attached detailed responses to the reviewers’ comments below. We believe the feedback has strengthened the manuscript and welcome further editorial suggestions.

Thank you again for your consideration of our manuscript.

Sincerely,

Jacob Bor ([jbor@bu.edu](mailto:jbor@bu.edu))

**Reviewer’s Comments to the Author**

**Reviewer #1:**

*I have several minor queries and comments listed below. There are no page numbers in the review package, so I have used the Heading, sub-heading and paragraph as a guide to where the comments were raised.*

**Response:** We have now added page and line numbers.

Comments:

*Introduction, paragraph 2:
As this is a South African national cohort, can the authorships add a ZAR equivalent value for the cost of inpatient HIV care?*

**Response:** We have now added the ZAR equivalent value to the cost of inpatient HIV care. In the introduction, we now write: “US$1000 (ZAR 18,500)”

*Methods: Study population:
Can the authors expand on the sentence “To maintain consistency, KwaZulu-Natal was excluded from all national-level analyses, because the province joined NHLS in 2010.”?
What analyses were KwaZulu-Natal excluded from?*

**Response:** KwaZulu-Natal (KZN) was excluded from all analyses presented in the main tables and figures. These analyses focus on South Africa at the national level. KZN was only included in the provincial-level analyses found in the supplementary tables and figures (Figure S1, Figure S2).

To improve the clarity of this sentence, we now write: “KwaZulu-Natal joined NHLS in 2010. To maintain consistency over time, KwaZulu-Natal was excluded from all national analyses but included in province-stratified analyses.” on Methods – study population.

*Does the NHLS National HIV Cohort (Table 1) include individuals from KwaZulu-Natal?
It may be helpful to include a breakdown of the number of individuals from each province of South Africa.*

**Response:** Yes, the NHLS National HIV Cohort (Table 1) includes individuals from KwaZulu-Natal. We have added a breakdown by province to the table.

*KwaZulu-Natal is the third-largest province in South Africa by population count. As per the latest estimates, approximately 2.1 million people living with HIV are located in KwaZulu-Natal out of the country’s total ~7.9 million PLWH. Excluding KwaZulu-Natal is a significant limitation of the study. This needs to be noted.*

**Response:** Thank you for pointing this out. Although this is a limitation of our study, we believe that the exclusion of KZN will not significantly impact our conclusions. When examining the provincial breakdowns of the number of individuals presenting and receiving HIV care (Figure S1, Figure S2), we observe that KZN follows a similar trend to the other provinces.

We added this note to the limitations. In the last paragraph of the discussion section, we now write:

“Fourth, KwaZulu-Natal was excluded from all national-level analyses as data were only available starting in 2010. In provincial breakdowns of individuals presenting and receiving HIV care (Figure S1, Figure S2), KZN follows similar trends as other provinces post-2010.”

*Methods: Analyses: Trends in hospital-based care and incident hospitalizations:
Do the authors know when the change in the ART regimens took place in South Africa?
Please add a reference & citation on the lessened toxicity of the one-pill regimen.*

**Response:** The changes in ART regimens took place on April 1, 2013. We have added this statement and added citations on the lessened toxicity of the one-pill regimen.

In Methods: Analyses: Trends in hospitalizations among patients established on ART, we now write: “HIV treatment regimens have become less toxic over time^33–35^. Since April 1, 2013, South Africa has offered fixed-dose combination (FDC) ART treatment, allowing patients to take just one pill, once per day^36^.”

*Methods: Analyses: Changes in the health status of hospitalized patients:
How was the threshold for hospitalization determined?*

**Response:** The definition of hospitalization is provided in the last paragraph of the section on “Measures”. The definition – tests taken on 2 or more days within a 7 day period at a hospital – was determined in order to exclude likely outpatient care at hospitals. Tests on just one day could be outpatient; tests more than a week apart could reflect a sequence of outpatient care. These were clinically-informed, heuristic decisions. We write:

“we defined “incident hospitalization” events as the presence of multiple lab tests (including CD4/Viral load as well as other blood work-up tests) taken on 2 or more days within a seven-day period at a hospital. We excluded hospitalizations occurring within the first 14 days of presentation or viral suppression, as these may have reflected the same care episode. We note that our lab-based measure of incident hospitalization is a proxy for an underlying clinical event (hospital admission) and we cannot rule out the possibility that the measure could capture some outpatient hospital care. To guide interpretation, we assessed whether patients experiencing “incident hospitalization” were in worse health than other patients, as would be expected if our measure captured significant patient morbidity. As described below, incident hospitalization was strongly correlated with worse health across a range of laboratory measures.”

*As this is a laboratory test-record-based dataset, were indicators of hospitalizations recorded in the lab records?*

**Response:** Unfortunately, there were no indicators of hospitalization recorded in the lab records. We have added this to the list of limitations of our data. In the second to last paragraph of the discussion section, we now write: “First, the NHLS database only included laboratory results and did not contain other information often available in clinical patient records, such as referral notes and hospital admission or discharge indicators.”

*Results: Paragraph 2:
On the statement “patients who were never hospitalized”, please add additional clarification, the patients were never hospitalized for HIV related treatments. The patients could have been hospitalized for other conditions, where a baseline HIV test was performed.*

**Response:** Our outcome of hospitalization was determined by the presence of laboratory tests in the data. “Patients who were never hospitalized” refers to HIV patients who never had any hospitalization event within our study period, as determined by our criteria of hospitalization (any hospital-based laboratory tests on at least two different days within a seven-day period). This definition (based on any laboratory test) means that hospitalization may/may not be for HIV reasons.

In our results section, we now write: “The health of patients who were hospitalized was substantially worse than the health of patients who never had a hospitalization event, with respect to their lab result values (Table 2).”

*Results: Paragraph 12:
Two points here, the first one is a bit more philosophical. “Additionally, nearly the entire rise in hospitalization occurred among patients who presented for care at hospitals.” – one can not be hospitalized if they are not at a hospital. Can the authors add some clarity here, can a patient be referred for hospitalization by a Clinic, without the hospital having to repeat the HIV tests?*

**Response:** We measure hospitalization events that occur after presentation to care. To exclude patients who were hospitalized at the same time they initially presented for care, we excluded events occurring within the first 14 days of presentation in our analyses. The distinction between hospitals and clinics pertains only to the location of the initial care presentation, and not to the subsequent hospitalization episodes.

We now clarify: “Additionally, nearly the entire rise in hospitalization occurred among patients who initially presented for HIV care at hospitals (**Figure S4**), suggesting that changes in clinical procedures within hospitals, not rising morbidity, may explain the apparent rise in hospitalizations.”

*Second comment, do the authors know how many patients first presented to a Clinic for HIV care, and subsequentially were referred to a Hospital for continued HIV care?*

**Response:** Although the initial ART rollout occurred in hospitals, clinics quickly became the primary point of routine HIV care in South Africa. To avoid drain on hospital resources, patients receive ART at outpatient clinics unless there are complications requiring hospital level care, including inpatient hospitalization. However, once stabilized, patients are referred to outpatient clinics.

*Discussion: Paragraph 4:
“Another potential explanation is that the success of the country’s treatment program and declining numbers of new patients entering care may have reduced congestion pressures at hospitals.” – I have two issues with this statement. Firstly, it directly contradicts the authors’ prior statement a few sentences earlier “Thus, there is a concern that hospitals remain overburdened and under pressure.”. Secondly, although the improvement of HIV care over time in South Africa indeed has had significant positive impacts on the health and wellbeing of PLWH. However, proposing that the increase in HIV-related hospitalization is due to increased hospital capacity is unfounded as no evidence pointing to the increased hospital capacity in South Africa is presented in the manuscript. This is also a siloed view of public care in South Africa. HIV care is but one of many disciplines of healthcare. The demand for the public healthcare system in South Africa continues to increase year-on-year. Therefore, this statement suggests a false viewpoint, unless the authors can present evidence for the contrary.*

**Response:** We have rephrased to indicate that these potential explanations for our findings are necessarily speculative and require further investigation. We have removed the reference to “supply-induced demand”. We agree with the reviewer that there is nothing to suggest that demand is falling. Rather, hospitals were likely forced to ration care to the sickest early in the ART rollout and later on were able to care for more PLHIV who needed hospital care.

First, we write: “raising concern that hospitals may remain overburdened and under pressure”. We add “may” as we do not present evidence that hospitals are overburdened, just that the numbers of HIV patients receiving hospital care have not declined.

Second, we write: “One possible, if speculative, explanation is that the success of the country’s treatment program, with improvements in patient health and declining numbers entering HIV care, may have reduced congestion pressures at hospitals. Patients hospitalized in 2015-2017 were hospitalized while in better health than previously, at least according to available laboratory measures. It is possible that hospitals had to ration HIV care to the sickest patients early in the study period, but later were able to allocate resources to marginally healthier patients leading to higher hospitalization rates as patients were able to access the care they needed. Further investigation and alternate data on hospital congestion are needed to test this hypothesis and investigate other explanations for the rise in hospitalization after 2013.”

*Grammar, abbreviations and consistency:
1. “In-patient” or “inpatient” & “out-patient” or “outpatient”, please pick one and be consistent.
2. Methods: Study context: “PHC”, please expand. This is the first use of this abbreviation.
3. Methods: Study context: Please add units for CD4.
4. Methods: Data source: “VL”, please expand. First use of the abbreviation and please be consistent with its use.
5. Results: paragraph 1: “In total 66%..” please add a comma: “In total, 66% …”.*

**Response:** We have fixed the grammar, abbreviations, and consistency as stated in points 1-5.

1. Replaced all “in-patient” to “inpatient”, and all “out-patient” to “outpatient”.
2. Expanded the abbreviation of primary health care (PHC).
3. Added units for CD4 counts (cells/μL).
4. Expanded the abbreviation of viral load (VL). Replaced all other used of “viral load” into the abbreviation “VL”.
5. Added a comma after “in total”.

**Reviewer #2:**

*Thank you for the opportunity to review this paper. Although its labelled revision 1, I was seeing it for the first time. The paper was generally well written and addresses an important issue around continuing HIV related morbidity and hospitalization in the era of widely available HAART. The so what of the paper could be better handled/ discussed. The paper didn't have page numbers or line numbers which made reviewing and referencing sections difficult. I have made more detailed comments below:*

**Response:** Thank you for taking the time to review this paper. We have now added page and line numbers to make reviewing easier.

*Abstract
- the authors refer to their database as novel. I don't agree. This NHLS cohort created from record linkage has been around for a while - as early as 2015/2016 and has been used to publish on different HIV related outcomes . Maybe its the first time they have used it to look at hospitalization as an outcome, but its certainly not novel*

**Response:** We have removed the words “a novel database:” when describing the NHLS HIV cohort on abstract, methods section.

*- why 2017? the data is already dated and so many events that could potentially affect hospitalizations among HIV infected have happened i.e. COVID-19 pandemic and DTG roll-out*

**Response:** We do not have access to data beyond 2017, however the data we have enable us to answer our key questions.

Our goal was to highlight trends in hospitalizations during an era of major HIV policy changes on HIV care in South Africa. During the 2004-2017 period, many major policy changes impacted HIV care in the country, including the ART rollout, the introduction of NIMART, and expanding the CD4 count thresholds for ART eligibility, among other changes. Since 2017, there have been fewer changes in HIV care (rollout of DTG notwithstanding), and the COVID era is rather idiosyncratic and out of scope.

We have added this to our limitations. In the last paragraph of our discussion section, we now write: “Fifth, due to limitations in data availability, we were unable to analyze policy changes beyond 2017, such as the DTG rollout, which may affect hospitalizations. However, the period of study captures most major HIV policy changes in South Africa, including the ART rollout, the introduction of NIMART, the expansion of CD4 count thresholds for ART eligibility, introductions of new ART regimens, and improved diagnostics for TB. Since 2017, there have been few major changes to HIV care, the rollout of DTG notwithstanding. Nevertheless, we caution against extrapolating these results to today's ART program.”

*- the outcome was determined from the variable facility type i.e. clinic vs hospital. How did the authors handle hospital based out-patient , ambulatory HIV services which may not be labelled clinics but function as such*

**Response:** Often such outpatient hospital-based clinics are recorded as separate clinics within the NHLS database, e.g. these are often referred to as “gateway clinics”. However, there may be cases where patients receive outpatient services at hospitals or at hospital-based clinics that are not separately coded within the NHLS database.

We handled this in two ways. First, we defined “hospital-based care” as any care occurring in a hospital, not just inpatient care. We now clarify:

“We defined an indicator for whether a patient was “receiving HIV care at a hospital” or “receiving HIV care at a clinic” in a given year by the presence of either a CD4 count or viral load result at that facility type in that year.”

Second, we define incident “hospitalization” in a way that limits misclassification of outpatient care. Specifically, we require lab tests on two separate days within a 7-day period at a hospital. Such a care-seeking pattern is unlikely for outpatient care.

*Introduction
- the authors didn't mention about NCD related comorbidities that are becoming increasingly common among individuals aging on ART as a potential factor in increasing hospitalization of PLHIV over time*

**Response:** Thank you for this suggestion. We added this paragraph to the introduction:

“Although ART has reduced hospitalizations associated with HIV and AIDS, longer life expectancy of PLWH has increased risk for non-communicable diseases (NCDs)^18–21^, including diabetes, hyperglycaemia, and renal insufficiency^21^. These comorbidities among older PLWH are associated with higher healthcare utilization^22^, increased hospitalization rates^23^, and excess mortality^24^.”

*- the authors refer to this database as novel- please see abstract for comment on this*

**Response:** We have removed the words “a novel database:” from the introduction section.

*Methods
Under data sources - the authors refer to the CD4 count as a measure of health at ART start . I think its best to say as a measure of immune status. Health is too non-specific*

**Response:** We now write “to measure immune status at ART initiation” under Methods, data sources.

*Under measures - why an ELISA for HIV when the bulk of HIV diagnoses are done by rapid HIV testing. What was the rationale for the ELISA. The same question applies to the PCR?*

**Response:** Entry to HIV care was defined using VL and CD4, following the South African national guidelines. PCR and ELISA were not used to define entry to HIV care but were used to infer hospitalization episodes along with all other laboratory tests.

*Why weren't TB related measures included when disseminated TB is the commonest opportunistic infection among PLHIV. Measures of disseminated TB could have been explored - eg CSF samples for TB,*

**Response:** Thank you for this comment. This was a limitation of our data. We did not have access to laboratory test data for TB for this study.

*As markers of incident hospitalization- why only blood work-up?*

**Response:** This was a limitation of the data we had available in the cohort. However, we now note this as a limitation. “We were also limited in the types of laboratory tests available for inclusion in the study.”

*Also was there an attempt to look at drug resistance data since ART failures are an important factor contributing to hospitalisations*

**Response:** We agree that examining drug resistance data would be of interest. Unfortunately, this is a limitation of the data available for this study.

*Analyses
- comparing risk of hospitalization in 2004 vs 2017 doesn't address the query I had about increasing risk of hospitalization with increasing age in the same individuals over time. Is it possible to include this type of analysis*

**Response:** When assessing risk of hospitalization over time for a given individual, it is very difficult to disentangle, age, calendar time, and time since ART initiation, each of which may be related to hospitalization risk.

That said, we are able to assess whether, within the whole cohort, the risk of hospitalization within 2 years of care-seeking varies over the study period for people of different ages. We have now added this analysis to the supplement and discuss it in the methods and results section.

We write in the methods section: “We also assessed annual trends in incident hospitalization by age groups: 18-39 and 65+”

And in the results section: “In an age-stratified analysis, PLWH over 65 years entering care after 2010 had an elevated risk of hospitalization compared to PLWH 18-39 years (**Figure S5**).”

*Results
- the authors found quite large numbers of people who had a CD4 count/ viral load and therefore assumed to have initiated HIV care or started ART. Was there an attempt to check with other data sources whether these numbers are plausible. SA has an estimated 5.8 million people on ART out of 7.4 million PLHIV. Does this mean over the years 2 million people plus have died from HIV?*

**Response:** In 2017, there were about 7M South African adults living with HIV, of which there were about 4M on ART (and should have a viral load); many of the rest of the people currently with HIV have sought care (and would have a CD4 count) but are not on ART. UNAIDS estimates that 1.8M adults in SA have died between 2004 and 2017. This puts the total number of adults with HIV during the period 2004-2017 at about 8.8M.

Our database includes 9.6M adults with a CD4 or VL from 2004-2017. About 4 million have a VL within the last 18 months of the database, very similar to the total number on ART. The total numbers in the NHLS cohort are in the realm of plausibility. That said, they may be a bit higher than expected due to care-seeking among people who migrated out of South Africa; because people may seek care under multiple names; and because gaps in Sensitivity of the linkage algorithm can split records into two.

We refer the reviewer to the the Cohort Profile for further details… <https://bmjopen.bmj.com/content/12/10/e066671>

*- in paragraph 5 of the results section, the authors present results on children when in the method state that they only included adults in the analysis.*

**Response:** Thank you for pointing this out. This was an error on our part. When we mentioned “children”, we were referring to adults aged 18-24. We have now made a correction to this. Instead of “children”, we now write “young adults”.

*Discussion
the so what of the paper is could be strengthened. In the discussion, the contribution of disengagement from care and drug resistance to hospitalization has not been discussed. As early as 2012/2013 a study from Cape Town found that half of HIV related admissions were among people who had disengaged form care
Any recommendations for policy or practice?*

**Response:** Thank you for this suggestion. We have now revised Figure 1D to show a plot describing the percentage of hospitalization that is “at presentation”; the % declines from 72% in 2004 to 27% in 2010 to 16% in 2015, indicating that a growing share of people receiving hospital-based care have previously sought care for HIV and are not new patients. We cannot, with the data we have, distinguish between patients who disengaged from care or experienced major adherence issues and patients who developed drug resistance.

We now write: “Our analysis reveals several successes of the national HIV program, but also some causes for concern. Even as fewer new patients entered care towards the end of the study period, a considerable proportion still presented with very low CD4 counts, consistent with findings from previous studies54. Low CD4 counts at entry to care are associated with a higher probability of hospitalization in these and other data55–57. Additionally, over time a growing share of hospital-based care was provided for PLHIV who previously entered care and may have disengaged from care or experienced adherence challenges. Previous research found that HIV-related hospitalization remains common despite the success of ART scale-up in South Africa10,11. We observed that the number of patients receiving care in hospitals flat-lined after 2013. At the same time, there was no increase in the number of hospital beds in South Africa 58, according to World Bank statistics, raising concern that hospitals may remain overburdened and under pressure.

“Lastly, we observed an increasing risk of incident hospitalization from 2013 onwards. This rise was particularly pronounced when adjusting for patient CD4 count and facility type at presentation. We believe it is unlikely that this rising trend reflects a substantial increase in patient morbidity – which has not been reported elsewhere. We conducted several robustness checks and were able to rule out explanations related to the dataset or definitions. One possible, if speculative, explanation is that the success of the country’s treatment program, with improvements in patient health and declining numbers entering HIV care, may have reduced congestion pressures at hospitals. Patients hospitalized in 2015-2017 were hospitalized while in better health than previously, at least according to available laboratory measures. It is possible that hospitals had to ration HIV care to the sickest patients early in the study period, but later were able to allocate resources to marginally healthier patients leading to higher hospitalization rates as patients were able to access the care they needed. Further investigation and alternate data on hospital congestion are needed to test this hypothesis and investigate other explanations for the rise in hospitalization after 2013.”

**Reviewer #3:**

*General*
*• This is a clear and well-written manuscript assessing temporal changes in hospital- vs primary care clinic-based care and incident hospitalisation within a very large and highly representative in South Africa from 2004 to 2017. While the data is no longer very recent, the authors position the importance of this topic well in the introduction, highlighting implications of hospitalisation both for individual health and the health system. For the most part, I have only minor comments for consideration by the authors.*

**Response:** Thank you for the comments.

*Methods:*
*• Subsection “data sources”, paragraph 3: Please consider whether the word “anonymized” is accurate in this case or whether pseudonymized, deidentified etc. might be more appropriate (under “ethical considerations”, the term “de-identified” is used). Though often used differently, “anonymized” implies that no linkage is even theoretically possible – i.e. that all direct and indirect links between identifiers and identifying information have been removed.*

**Response:** We now write “de-identified” instead of “anonymized” on data sources, paragraph 3.

*• Subsection “measures”, paragraph 1: The abbreviation “CD4” is used repeatedly before being introduced here.*

**Response:** We have moved the definition to the first occurrence within the main text. As it is a widely used abbreviation, we do not define it in the abstract.

*• Subsection “measures”, paragraph 3: “We excluded hospitalizations occurring within the first 14 days of presentation or viral suppression, as these may have reflected the same care episode.” This is not entirely clear to me and appears to by default exclude hospitalization at presentation. If I understand correctly, the authors may consider clarifying that this manuscript addressed incident hospitalisation among people already in care for HIV (/in pre-ART care), not incident hospitalisation related to late presentation. This appears to be contradicted by the tested association between health (CD4 count) at presentation and risk of hospitalization, so I apologize to the authors if I misunderstood this.*

**Response:** The reviewer is correct. The definition of “hospitalization” required only having lab tests on two separate days within a seven day period. However, in our analyses of incident hospitalizations following entry into care or viral suppression, we excluded hospitalizations occurring in the first 14 days. The rationale was that we sought to understand temporal trends in rates of hospitalization among persons entering care or established on ART, after controlling for where the person presented for care.

We now write: “Finally, we defined “hospitalization” episodes as the presence of multiple lab tests (including CD4/Viral load as well as other blood work-up tests) taken on 2 or more days within a seven-day period at a hospital. Individuals could have multiple hospitalizations. When assessing incident hospitalizations following entry into care or viral suppression, we excluded hospitalizations occurring within the first 14 days of presentation or viral suppression, as these may have reflected the same care episode.”

*• The authors could briefly mention how missing data and multiple hospitalizations were handled.*

**Response:** The NHLS Cohort includes all laboratory tests taken within South Africa’s public sector HIV program. (With the exception of KwaZulu-Natal pre-2010.) Thus there are no “missing data” within the dataset. It is possible that underlinkage could result in some lab tests not being assigned to the correct patient. In this case, from a patient perspective, there may be some missing data, which would bias downwards our estimates of hospitalization. The extent of missing data is very small however (~6% based on a prior validation study).

We now write in the limitations section: “Second, as with any large linked administrative database, our data are not impervious to linkage errors. The NHLS National HIV Cohort obtained high sensitivity and PPV in a validation study^29^ and thus our findings should be fairly robust to linkage error.”

Individuals could have multiple hospitalizations. We now write in the methods section: “we defined “hospitalization” episodes as the presence of multiple lab tests (including CD4/Viral load as well as other blood work-up tests) taken on 2 or more days within a seven-day period at a hospital. Individuals could have multiple hospitalizations.”

*Results:
• Table 1: In the caption, “20018” should probably read “2017”.*

**Response:** Thank you for noticing the typo. We now write “2017” instead of “20018”.

*Is it correct that there was no missing data for the displayed variables (the authors may wish to briefly address missing data in the methods)?*

**Response:** See the above comments on how missing data was handled.

*For hospitalizations up to or more than 6 nights, please indicate more clearly that the denominator is all people with hospitalization, not all people in the dataset.*

**Response:** We added “% denominator for duration of hospitalization is out of all patients with any hospitalization.” Under the “Notes” for table 1.

*I suggest additionally showing the proportion of all people in the dataset who had a hospitalization.*

**Response:** We added a line in Table 1 that shows the proportion of all people in the dataset with ≥1 hospitalization. The denominator for this hospitalization measure excluded PLWH accessing care in KZN and entering care after 2015. We added “% denominator for the proportion of PLWH with ≥1 hospitalization is N=6,536,201, after excluding PLWH accessing care in KZN and entering care after 2015.” Under the “Notes” for table 1.

*• Paragraph 5 (description of figure 2): While it is clear from context and is likely known to most readers, I suggest stating explicitly which age groups are considered “most affected by HIV”.*

**Response:** We now write: “Over the period of study, the risk of hospitalization declined the most in adults 25-45 years old, the age groups most affected by HIV”.

*• Figure 4 C-F, Figure 5B: I find these panels hard to read especially as similar colours are used for non-consecutive years. Perhaps a colour gradient could be used such that consecutive years have more similar colours.*

**Response:** We used a color gradient going from green-to-yellow-to-red for each year. We think it is the overlap between years that makes these figures a bit difficult to read and appreciate the reviewer flagging this for us. To make the figures easier to look at, we have tried reducing the number of years shown from 12 (2004, 2005,…,2015) to 4 (representing 2004, 2008, 2012, 2015). While we tend to prefer the original figures, we welcome further feedback from the reviewer and editor regarding visual clarity. We show an example of a revised Figure 4D below.

*• “After adjusting for changes in patient characteristics (Figure 2A, yellow line), the risk of hospitalization was nearly as high in 2015 and in 2004” I believe this should be Figure 3A.*

**Response:** That is correct. Thank you for pointing this out. We now write “(Figure 3A, yellow line)”.

*Discussion:
• “Our regression models indicate that CD4 count at care presentation and facility at presentation, explained almost half of the trend in hospitalization risk.” Here and elsewhere, “explained” appears to me to imply causality. However, it is not clear to me that the methods justify causal statements rather than statements of association. Regarding facility at presentation, do the authors believe that presentation at a clinic is directly causative of a lower risk of hospitalization (e.g. because “patients receiving outpatient care in hospitals may have been more likely to be hospitalized due to the availability of inpatient services on site” as noted in the methods), or that both presentation at a clinic and lower risk of hospitalization have a shared underlying cause (e.g. better health beyond what can be adjusted for with CD4 count)? In the former case, there could be concern that clinic presentation reduces the likelihood of receiving a required hospitalization.*

**Response:** The reviewer makes good points here regarding multiple potential interpretations of the data. Indeed, adjusting for facility at presentation captures both where care is available and aspects of patient health at presentation. To avoid implying causality, we now write:

“Our regression models indicate that improvements in patient CD4 counts at presentation and the increase in care-seeking at clinics statistically explained almost half of the decline in hospitalization risk.”

*• The authors draw some conclusions for HIV care today from this data – e.g., that “there may still be opportunities for earlier case-finding and reduction in incident hospitalizations in PLWH”. While I certainly agree, it may be appropriate to briefly note as a limitation that follow-up ended in 2017 and extrapolations to today’s ART programme are difficult.*

**Response:** We added this statement to our limitations. On the last paragraph of our discussion section, we write:

“Fifth, due to limitations in data availability, we were unable to analyze policy changes beyond 2017, such as the DTG rollout, which may affect hospitalizations. However, the period of study captures most major HIV policy changes in South Africa, including the ART rollout, the introduction of NIMART, the expansion of CD4 count thresholds for ART eligibility, introductions of new ART regimens, and improved diagnostics for TB. Since 2017, there have been few major changes to HIV care, the rollout of DTG notwithstanding. Nevertheless, we caution against extrapolating these results to today's ART program.”
